# Predicting Behaviour Patterns in Online and PDF Magazines with AI Eye-Tracking

**DOI:** 10.3390/bs14080677

**Published:** 2024-08-05

**Authors:** Hedda Martina Šola, Fayyaz Hussain Qureshi, Sarwar Khawaja

**Affiliations:** 1 Oxford Centre For Applied Research and Entrepreneurship (OxCARE), Oxford Business College, 65 George Street, Oxford OX1 2BQ, UK; 2 Institute for Neuromarketing & Intellectual Property, Jurja Ves III spur no 4, 10000 Zagreb, Croatia; 3 Oxford Business College, 65 George Street, Oxford OX1 2BQ, UK; fayyaz.qureshi@oxfordbusinesscollege.ac.uk (F.H.Q.); advice@oxfordbusinesscollege.ac.uk (S.K.)

**Keywords:** neuromarketing, consumer-behaviour research, AI eye tracking, AI, EEG, consumer neuroscience, college magazine research

## Abstract

This study aims to improve college magazines, making them more engaging and user-friendly. We combined eye-tracking technology with artificial intelligence to accurately predict consumer behaviours and preferences. Our analysis included three college magazines, both online and in PDF format. We evaluated user experience using neuromarketing eye-tracking AI prediction software, trained on a large consumer neuroscience dataset of eye-tracking recordings from 180,000 participants, using Tobii X2 30 equipment, encompassing over 100 billion data points and 15 consumer contexts. An analysis was conducted with R programming v. 2023.06.0+421 and advanced SPSS statistics v. 27, IBM. (ANOVA, Welch’s Two-Sample *t*-test, and Pearson’s correlation). Our research demonstrated the potential of modern eye-tracking AI technologies in providing insights into various types of attention, including focus, engagement, cognitive demand, and clarity. The scientific accuracy of our findings, at 97–99%, underscores the reliability and robustness of our research, instilling confidence in the audience. This study also emphasizes the potential for future research to explore automated datasets, enhancing reliability and applicability across various fields and inspiring hope for further advancements in the field.

## 1. Introduction

In the contemporary digital era, content consumption has substantially transformed, primarily owing to the proliferation of online magazines and the ubiquitous utilisation of PDF versions. Previously, college magazines were exclusively confined to print media; however, they have since migrated to the digital domain to expand their reach and deliver a more immersive reading experience. Analysing users’ reading habits is imperative to our understanding of how they interact with online and PDF college magazines.

Eye-tracking technology is commonly employed in research on visual attention and user behaviours. This technology captures individuals’ gaze patterns as they interact with digital content, permitting researchers to discern which elements draw attention, how long they maintain focus, and the order in which they explore information. Eye-tracking software has emerged as a potent tool for uncovering the intricate aspects of user interaction, illuminating how readers navigate, comprehend, and engage with the content [1]. Eye-tracking technology captures and examines eye movements to understand visual attention and cognitive processes [2]. This technology has diverse applications in various fields, such as psychology, marketing, and user experience design.

In the context of online and PDF college magazines, eye-tracking software can provide valuable insights into user behaviour, enabling publishers to optimise the content layout, design, and overall user experience [3]. College magazines have historically served as platforms for students to showcase their creativity, share accomplishments, and display the energy of campus life [4]. These magazines initially emerged as print publications, offering readers tangible and tactile experiences [4]. However, the advent of digital platforms and the Internet has resulted in the transition to college magazines, presenting opportunities and challenges [5]. The shift from traditional print to digital formats in college magazines reflects contemporary readers’ changing preferences. Online magazines and PDF versions provide multimedia integration, hyperlinks, and dynamic content, enhancing the reading experience [6]. Online college magazines now incorporate multimedia elements, interactive features, and global reach, thus surpassing the limitations of print. By contrast, PDF formats preserve a resemblance to the traditional magazine layout while offering a digital reading experience. Understanding how readers navigate and engage with content in these formats is crucial to enhancing the design and delivery of college magazines [5]. The predictive capabilities of eye-tracking software provide valuable insights into user preferences and the effectiveness of content presentation [7]. When applied to college magazines, eye-tracking software allows for meticulously examining how readers consume content. Researchers can pinpoint areas of significant interest, track reading paths, and assess the influence of design choices on user engagement [8]. Eye-tracking technology can yield valuable insights into the interaction of students with educational content, thereby facilitating the creation of more impactful and compelling learning experiences. Furthermore, it can provide crucial insights into how individuals comprehend written content and focus on the visual elements relevant to online education and digital publications [9]. In addition, eye tracking has been employed to evaluate the magazine layout, offering valuable insights into how individuals perceive and understand displayed information. Moreover, studies have demonstrated the ability of eye-tracking to assess reading comprehension.

These advantages illustrate the capacity of eye-tracking software to enhance the design and distribution of instructional content in online and PDF college magazines [10]. Eye-tracking software can significantly improve the user experience of college magazines by providing valuable insights into how readers engage with content. This technology can contribute to improving user experience in several ways.

1. Eye-tracking data can provide valuable insights into readers’ natural gaze patterns, indicating where their attention is focused and how they navigate the content. By utilising this information, designers can optimise the layout of college magazines by placing critical information, headings, and multimedia elements in areas where users are most likely to look first, resulting in a more intuitive and user-friendly reading experience [5].
2. Understanding the hierarchy of visual elements is crucial for capturing and maintaining the user’s attention. Eye tracking can help identify the elements that users perceive as more prominent or essential, enabling designers to adjust the visual hierarchy accordingly to guide readers through the content in a way that aligns with the intended narrative or informational flow [9].
3. Eye-tracking technology can provide insights into how users consume textual content. By analysing reading patterns, designers can enhance the readability of college magazines. This includes optimising font sizes, line spacing, and paragraph lengths to ensure a comfortable reading experience. It also helps identify optimal placements for pull quotes, captions, and other text elements [11]. Incorporating multimedia elements, such as visuals, videos, and interactive features, is common in college publications. Eye-tracking data can offer valuable insights into how users interact with these components. Designers can optimise the placement and visibility of multimedia aspects to enhance the user experience and provide engaging, informative content that caters to user preferences [12]. Eye tracking allows designers to differentiate between user behaviour on online platforms and PDF formats. This information is crucial for effectively tailoring the design of each medium. For instance, online readers may display distinct gaze patterns owing to interactive elements, while PDF readers may follow a more traditional reading flow. By understanding these differences, designers can create seamless and enjoyable experiences across various formats [13].
4. Responsive design is essential for delivering an optimal user experience across different devices and screen sizes. Eye-tracking data can provide valuable insights into how users interact with content on various devices. This information can be used to create responsive layouts that adapt to different screen sizes, ensuring that the college magazine remains visually appealing and user-friendly on both desktop and mobile devices [13].
5. Eye tracking enables usability testing by offering real-time information about user interactions with prototypes. This tool allows designers to observe user gaze patterns and identify potential usability issues. Through this iterative design process, continuous improvement based on user feedback is possible, resulting in a more refined and user-centric college magazine [9].

The Internet has revolutionised college magazines by introducing interactive and dynamic features. By incorporating multimedia elements, such as videos, animations, and hyperlinks, the overall reading experience is enhanced. However, these additions present challenges in terms of visual attention. Eye tracking is a helpful technique for understanding readers’ preferences; how they engage with textual content, multimedia features, and interactive elements; and how these factors contribute to comprehension and engagement. This technology is widely used in market research, psychology, and user-experience design. It employs various techniques, including infrared cameras that detect the reflection of light from the eye and computer algorithms that analyse the gathered data. Eye-tracking technology has transformed market research, psychology, and human–computer interaction. However, eye tracking in online magazines presents a unique challenge owing to scroll-based navigation. Unlike the page-turning experience of PDFs, readers of online magazines may scroll quickly or linger in specific sections. While PDF college magazines lack the dynamic features of their online counterparts, they retain a familiar layout reminiscent of traditional print publications. Studies on PDFs using eye tracking can reveal whether readers follow a linear reading path, focus on specific sections, or exhibit a pattern similar to print reading behaviour. Analysing gaze data in PDFs helps to assess the effectiveness of static layouts, typography choices, and the integration of images. The constraints associated with PDFs in predicting user engagement based solely on gaze patterns are substantial because of the shortage of interactive components. Unlike digital magazines, PDFs do not provide immediate feedback between the user’s gaze and interactive elements, which necessitates contextual interpretation of gaze data in light of the intrinsic limitations of the format. Comprehending how users interact with college magazines is paramount for content creators, designers, and educators as the digital environment transforms. This comparative study endeavours to untangle the complexities of visual attention by utilising neuromarketing eye-tracking software, offering invaluable insights into the merits and limitations of this technology in online and PDF college magazines. By cultivating a nuanced comprehension of user behaviour, this study aims to facilitate well-informed design decisions that elevate the overall reading experience and optimise the impact of college magazines in the digital age.

Additionally, this comparative study aimed to evaluate and assess the various eye-tracking software tools utilised in online and PDF college magazines. This assessment intended to pinpoint the strengths, weaknesses, and potential consequences of integrating these tools to enhance the overall efficacy of digital college magazines.

Hypothesis: How reliable is AI eye tracking in predicting how online content affects readers’ attention patterns?

## 2. Literature Review

### 2.1. Consumer Behaviour-Prediction Software

Integrating innovative technologies like eye-tracking software can enhance consumer-behaviour prediction using AI. As highlighted in various studies [14,15], AI techniques are crucial in reshaping consumer-behaviour analysis by leveraging data insights. The use of AI in predicting consumer behaviour is further supported by the increasing reliance on digital technologies and online communities [14]. Additionally, applying big data analytics and machine learning in social media platforms aids in predicting consumer behaviour based on various parameters and criteria [15]. Furthermore, the review of AI-based solutions in sensory and consumer studies emphasises the importance of AI in efficiently exploring and correlating data to benefit the food industry and consumers [16]. Researchers can potentially gain deeper insights into consumer-behaviour patterns and preferences by incorporating eye-tracking software into AI models. AI technology is crucial in accurately predicting consumer behaviour through various advanced models and techniques. Studies have shown that AI can significantly impact consumer behaviour by enhancing e-payment systems, stimulating consumer sentiments, and improving personalised services [17]. Deep learning models, particularly ensemble architectures, have been developed to efficiently classify complex consumer-behaviour variants with high precision, outperforming traditional machine-learning models [18]. Additionally, integrating AI models with marketing data using decision-making models, such as decision trees and genetic algorithms, has proven to predict consumer behaviour in digital and physical shopping environments, achieving classification accuracies above 90% [19]. Furthermore, research indicates that factors like trust, attachment, empathy, loyalty, and gender characteristics play significant roles in shaping consumer–AI relationships, highlighting the importance of understanding these dynamics for optimal integration of AI in consumer interactions [20].

Moreover, AI models that combine marketing data and computer science methods, such as decision trees and genetic algorithms, are essential in classifying users’ needs, predicting consumer behaviour, and aiding decision-makers in understanding client needs in digital and physical shopping environments [19].

### 2.2. Introduction to AI Eye Tracking in Consumer-Behaviour Prediction

AI-driven eye-tracking technology is crucial in predicting consumer behaviour by analysing visual attention patterns and cognitive processes. Studies have shown that eye-tracking data, coupled with machine-learning algorithms like K-means clustering [21], can help identify consumer preferences and predict decision-making processes based on visual attention [21]. Additionally, the accuracy of gaze data from eye trackers can be enhanced through machine learning models, enabling the detection and prediction of gaze error patterns under various conditions [21]. Furthermore, AI technologies, such as deep neural networks (DNNs), have been utilised to analyse consumer facial expressions and predict psychological behaviour, leading to personalised product recommendations and high consumer satisfaction rates [22]. Integrating AI technology in e-commerce businesses has also been shown to significantly impact consumer behaviours by enhancing accuracy, interactive experiences, and perceived values [17]. AI leverages eye-tracking technology to understand consumer behaviour by detecting specific areas of interest in advertising [23]. This technology enables the accurate and objective measurement of consumers’ attention, which is a fundamental variable in shaping attitudes and behaviours towards products and advertisements [23]. By analysing eye movement, fixations, and gaze patterns, AI systems can identify cognitive processes related to visual attention when consumers interact with products, such as choosing a shampoo brand [24]. Through clustering machine-learning approaches like K-means, AI can process and analyse large eye-tracking datasets to predict consumer preferences and behaviours based on visual attention attributes [24]. Integrating eye-tracking data with AI systems allows for a deeper understanding of consumer preferences and decision-making processes, enabling companies to tailor their marketing strategies and product designs accordingly.

An AI eye-tracking analysis involves several vital steps to ensure the accurate and efficient tracking of eye movements. Initially, the process begins with the image-capturing step, where a camera captures the facial image, including the eyeball, and light from a source forms a corneal reflex point on the eyeball, which is then reflected and captured [25]. The next step involves acquiring combined characteristic data, such as pupils, eyelid shape, and head posture, which are analysed using machine-learning methods to train a classifier with strong recognition performance, resulting in an eye-movement pointer [26]. The data are then coded into sequences of fixations and saccades using automated rules or advanced models, like the beta-process vector auto-regressive hidden Markov model (BP-AR-HMM), which provides a statistical model for eye-movement classification and identifies various eye-movement categories [27]. Deep learning-based algorithms, particularly convolutional neural networks (CNNs), are employed to classify and predict different aspects of eye movement and gaze estimation [28,29,30]. These enable the processing of large datasets collected through eye-tracking experiments, allowing for the identification of salient regions on interfaces, information presentation methods, and even user nationalities based on eye movements [29,30], considering different head poses and illumination conditions and ensuring high accuracy in detecting the gaze point [28]. Additionally, AI models can be compared with actual eye-tracking measurements to assess their accuracy and applicability in scientific research and business scenarios, providing valuable insights into the effectiveness of different measurement methods and their respective use cases [31]. By leveraging AI and deep learning algorithms, researchers can enhance the understanding of visual and cognitive processes; optimise user interfaces; and extract meaningful insights from eye-tracking data for various applications in academia, technology, marketing, and beyond. The visual-line information is then transformed and corrected for detection errors caused by individual differences in the eyeball, ensuring precise gaze tracking [25]. Finally, the results are validated by comparing AI-predicted eye-tracking data with actual measurements, such as those obtained from devices like the Tobii X120, to ensure the reliability of the AI model in both scientific research and real business applications [31]. This comprehensive approach leverages machine-learning, statistical-modelling, and deep-learning techniques to achieve a robust and accurate eye-tracking analysis.

### 2.3. Attention Patterns in Online vs. PDF Formats

Attention patterns in online and PDF formats exhibit distinct characteristics influenced by various factors, including content curation, user behaviour, and technological advancements. Online platforms, mainly those driven by user-generated content, often serve as attention bridges, facilitating the flow of attention across different sites and formats. In contrast, curatorial websites act as attention hubs, centralising user focus on specific content [32]. Implementing attention mechanisms in recurrent neural networks and self-attention models, such as the Routing Transformer, has significantly enhanced the efficiency and effectiveness of processing online content, enabling real-time attention computation, and reducing complexity [33,34]. These advancements are crucial for tasks like sentence summarisation, machine translation, and online speech recognition, which demand dynamic and sparse attention patterns to manage large volumes of data efficiently [33,34]. In contrast, PDF formats, often used for documents like invoices, reports, and forms, require sophisticated models like GraphDoc, which employs a multimodal graph attention-based approach to understand and analyse diverse document layouts and formats [35]. This model integrates textual, visual, and positional features to enhance document comprehension, demonstrating the importance of context-specific attention mechanisms in handling static content. Additionally, the spread of misinformation online underscores the need for an attention-based design to influence user decisions and improve task effectiveness, highlighting the psychological factors that affect information sharing on social media [36].

The differences in attention patterns between online and PDF formats can be significant, as evidenced by various studies. For instance, a study comparing multimedia information (MMI) with printed participant information sheets (PISs) found that participants rated multimedia as more straightforward to understand and were more likely to evaluate them positively. However, multimedia did not significantly increase recruitment or retention rates in clinical trials [37]. This suggests that multimedia formats can enhance comprehension and engagement. Similarly, research on online courses during the COVID-19 pandemic indicated that courses combining text and commentary attracted the most attention. In contrast, those using only text or commentary quickly lose the listener’s attention [38]. This aligns with findings from a study on social media recruitment, where the conversion rate from clicks to actual consent was low, but the cost-effectiveness and reach of online ads were notable [39]. Additionally, a study on ADHD behaviours on Twitter revealed that users with ADHD were more active in posting tweets, especially at night, and felt more intense emotions, suggesting that online platforms can capture more dynamic and emotionally charged interactions compared to static formats like PDFs [40]. In the context of data collection, digital methods were found to be convenient and reliable. However, some paper-based methods were more productive, indicating that familiarity and ease of use can influence attention and productivity [41]. Furthermore, research on the impact of funding on academic citation and social attention showed that funded studies, often published in open-access formats, received significantly more citations and social media mentions, highlighting the broader reach and impact of online formats [42]. The study on physical activity during the pandemic also noted that children exhibited healthier behaviour patterns when attending school in person compared to remote learning, suggesting that the information delivery format can affect engagement and behaviour [43]. Lastly, advancements in self-attention models like the Routing Transformer, which efficiently handles large data sequences, demonstrate the potential for online formats to manage and analyse complex information more effectively than traditional methods [33]. These studies underscore that online formats, particularly multimedia elements, enhance engagement, comprehension, and reach. In contrast, PDF formats may be more familiar but less dynamic and interactive.

### 2.4. Related Work

AI-driven eye-tracking technology has shown significant potential in predicting human behaviour, including the reading habits of magazine readers. Research has demonstrated that eye-movement data can be effectively utilised to understand decision-making processes and cognitive engagement. For instance, deep learning and support vector machine classification methods have successfully identified participants’ decision strategies in economic games by analysing their gaze behaviour, suggesting similar applications in reading contexts [44]. Additionally, machine-learning pipelines have been developed to detect developmental dyslexia through eye-tracking data, achieving high classification accuracy across different languages and experimental setups, indicating the robustness of such models in diverse reading scenarios [45]. In the realm of human behaviour recognition, hierarchical patch descriptor (HPD) and approximate locality-constrained linear coding (ALLC) algorithms have been employed to achieve high accuracy in recognising human actions, which could be adapted to analyse reading patterns [46]. Furthermore, AI models have been used to estimate visual acuity from optical coherence tomographic images, demonstrating the capability of AI to process complex visual data and make accurate predictions, which could be extended to understanding how readers interact with magazine content [47]. Eye-tracking has also been utilised to study the cognitive processing of feedback in educational games, revealing how different types of feedback are processed and utilised by learners, which could inform the design of interactive magazine content to enhance reader engagement [48]. Moreover, AI-assisted image-processing algorithms have been developed for precise measurements in medical contexts, such as ptosis evaluation, showcasing the precision and applicability of AI in analysing detailed visual data [49]. The study of subjective preferences in saccadic eye movements has revealed individual differences in gaze behaviour, which could be leveraged to personalise magazine content based on reader preferences [50]. Finally, the growing integration of AI into daily life and the importance of AI literacy emphasise the need for public awareness and understanding of these technologies, which will be crucial as AI-driven eye-tracking becomes more prevalent in consumer applications, including magazine readership analysis [51]. These studies illustrate the multifaceted applications of AI and eye-tracking in predicting and enhancing human behaviour, particularly in magazine reading.

## 3. Materials and Methods

We conducted a study in which we randomly selected three college magazines that were all geographically related to the Oxford area, with two being online publications and one in PDF format. The magazines we tested were *Hybrid Magazine*, written by students from Oxford Brooke University; *The Oxford Student*, written by students from Oxford University; and *OxConnect*, written by students from Oxford Business College.

We utilised a neuromarketing eye-tracking AI prediction software (Predict, version 1.0.) to evaluate the user experience of visiting websites and reading magazines. This software uses an algorithm developed with Stanford University, merging consumer psychology theory with neuroscience tools and insights. The software was built on one of the world’s largest consumer neuroscience databases (n = 180,000) with eye tracking (Eye Tracker: Tobii X2-30), comprising 100 bn+ consumer-behaviour data points, in reference to their total database. The number of complete databases confirms how relevant this software is and why we have to decide to use it, as it explains how the models are built, which models are highly predictive, and how the company will continue to expand on their models. The Predict model database consists of eye-tracking, EEG, and implicit-response data, but so far, the algorithm has only been built on the eye-tracking recordings. The eye-tracking recordings on sample size n = 180,000 were taken globally in 15 different consumer contexts and trained an encoder–decoder architecture (ConvNext as a pre-trained encoder) [52,53], where the database expanded monthly, which is yearly upgraded with 50,000+ participants and extended in various consumer contexts, as the AI learns each time that research has been performed. As this software was built in correlation with Stanford University, the provider has secured a predicate accuracy rate of 97–99% for attention, the highest in the industry. The model was designed to measure several aspects of user experience, including attention (start-end), which predicts the focal points of consumers during the initial and concluding 2 s of a 5-second exposure, an engagement metric to predict reader excitement and immersion when reading the magazine; focus, which explores the attentive readers’ attention in all tested magazines; clarity, which predicts how clear readers will find the tested magazines; and cognitive demand, which is highly aligned with cognitive load, which measures how much information viewers have to process while reading the magazines (the complexity of the image).

Measuring focus while reading to predict readers’ attention involves various cognitive and motivational factors. Awadh et al. and Lusnig et al. suggest that visual attention span is a significant predictor of reading fluency and comprehension [54,55], while Kobayashi and Kawashima introduce a text layout that manipulates visual attention by fading out characters, which improves comprehension by guiding the reader’s focus to the beginning of paragraphs [56]. This suggests that manipulating text layout can also effectively measure and direct readers’ focus. The focus score identifies areas where viewers’ eyes are likely to be concentrated, indicating heightened focus or scattered across the content, suggesting a more dispersed or distracted attention. To predict CD, the software relies on mathematical foundations that guarantee CD’s scores display reliability across diverse scenarios, accurately reflecting the cognitive demand of processing visuals. The software has been constructed upon industry-specific benchmarks for cognitive demand. It has evaluated scores based on the type of images, including in-clouding vertical ones such as websites, print ads, banners, and horizontal photos. This functionality makes the software highly useful in various neuromarketing research [52]. To conduct the study, we documented all three magazines—*Hybrid Magazine*, *The Oxford Student*, and *OxConnect*—by creating separate videos for each publication. The primary aim of these videos was to obtain a genuine user experience while browsing the three magazines’ websites. The videos could not be recorded for the same duration due to the varying amounts of content in each publication. Additionally, online magazines are not read in the same manner as PDF magazines, which influenced the decision to record the videos in the most precise time possible for online magazines. On the other hand, PDF magazines were recorded longer, as they are more challenging to read. The videos were prepared at high resolution (72.7 MB) and were recorded at the following time points: *Hybrid Magazine* (0:01:03 s), *OxConnect* (0:01:53 s), and *The Oxford Student* (0:59 s). By preparing the content, we could capture the listed parameters, such as cognitive demand, focus, clarity, engagement, and attention, while also analysing attention dynamics throughout the video. This enabled us to measure attention intensity and identify specific moments or sequences during which readers engaged with the magazines, either captivating or losing their focus. Ultimately, this approach allows us to test our hypotheses. The focus score identifies areas where viewers’ eyes are likely to be concentrated, indicating heightened focus or scattered across the content, suggesting a more dispersed or distracted attention. This software is grounded in a unique floating window operation that spans 2 s, ensuring precise, human-like attention behaviour predictions. By constantly analysing the shifting visual landscape of a video, this software can highlight those moments and elements most likely to seize a viewer’s gaze. This technique effectively captures the nuanced lags in human attention, ensuring a robust alignment of the model’s predictions with actual human eye movement patterns in video contexts (the related neuromarketing data can be found in Appendix H). The analysis for this study was conducted using the programming language R version 2023.06.0+421 and advanced SPSS statistics, version SPSS 27 (ANOVA, Welch’s Two-Sample *t*-test, and Pearson’s correlation) from the neuromarketing data gained from research. As per the neuromarketing research tool, scores ranging from 0 to 24 indicate low focus, indicating that numerous elements compete for attention. In contrast, scores ranging from 75 to 100 indicate high focus, suggesting that a single or few narrow areas attract the most attention and are more likely to be noticed. Similarly, scores ranging from 0 to 24 for CD suggest that the information is too easy to process, potentially reducing viewing time. Conversely, scores ranging from 75 to 100 indicate high complexity in the information, potentially overwhelming the viewers.

## 4. Results

After conducting Welch’s Two-Sample *t*-tests for cognitive demand (as detailed in Appendix A, Appendix B and Appendix C) and focus (as detailed in Appendix D, Appendix E and Appendix F) between each pair of magazines and a one-way ANOVA test for all three magazines, we discovered statistically significant differences among the magazines (as shown in Table 1, Table 2, Table 3 and Table 4). Following this discovery, we wanted to delve deeper into our analysis by incorporating benchmark indices specific to the industry and examining how content and its positioning can influence user experience.

### 4.1. Focus and Cognitive Demand

Our metrics suggest that cognitive demand scores, which gauge the amount of information that viewers must process, fall within a high range (75–100), indicating that the complexity of the content may overwhelm readers. Conversely, scores within the low range (0–24) suggest that the content may be too straightforward, potentially leading to shorter viewing times. Similarly, the focus metric, which assesses the degree of focused attention in the content, displays high scores (75–100) when several items competing for attention or a narrow range of items are most captivating. Low scores (0–24) indicate that multiple items vie for attention, making it difficult for viewers to concentrate.

**Figure 1:**
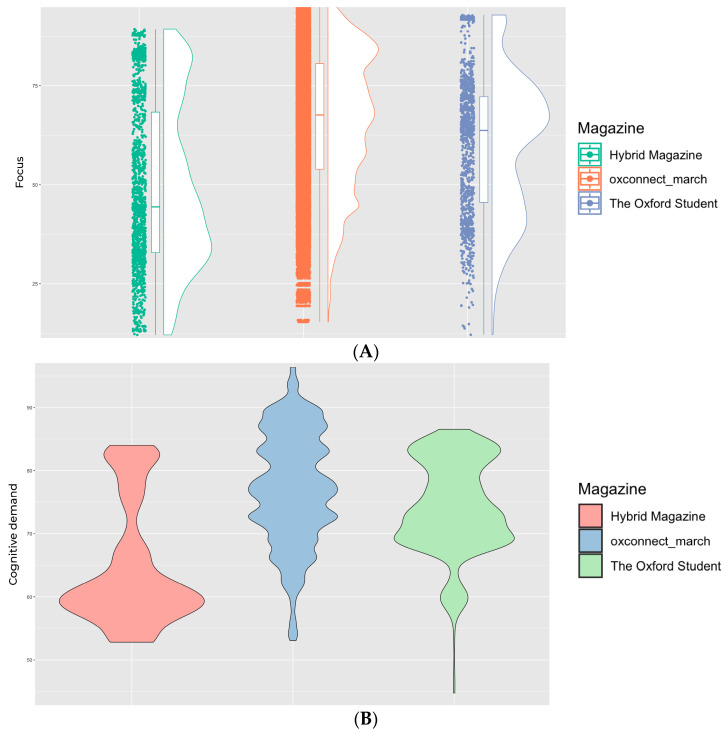
(**A**) Difference in focus for all 3 magazines. (**B**) Difference in cognitive demand for all 3 magazines.

**Figure 2:**
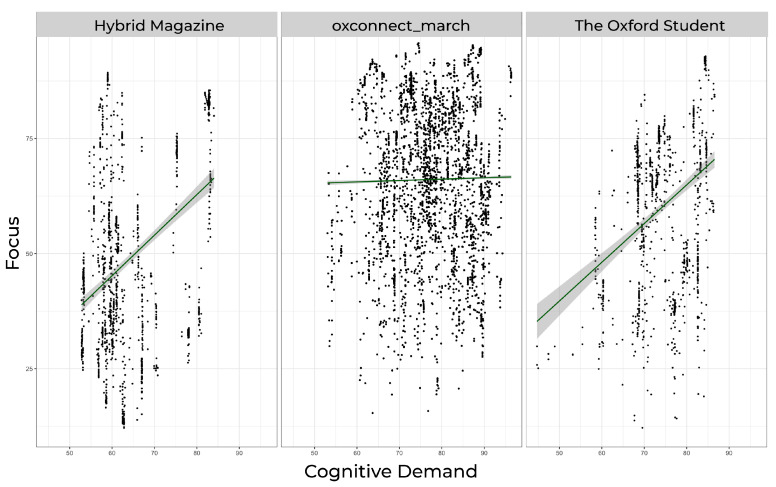
Linear relationship between cognitive demand and focus for all 3 magazines.

**Figure 3:**
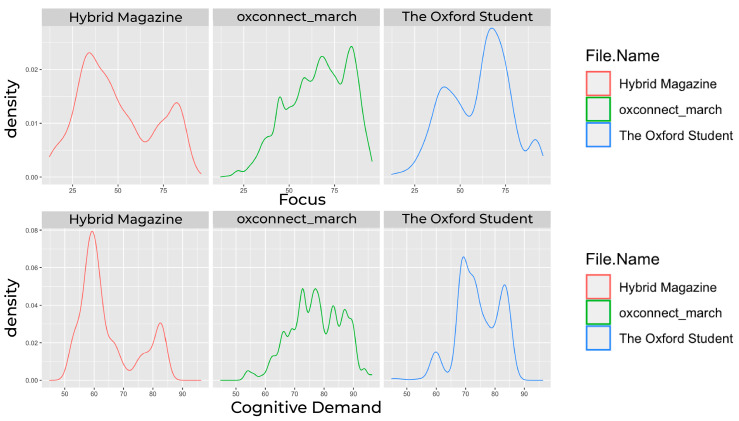
Focus score distributions for all the magazines with respect to each other (**top**). Cognitive demand score distributions for all the magazines with regard to (**bottom**).

Our findings show that both online magazines, *Hybrid Magazine* and *The Oxford Student*, fall outside the high zone for average cognitive demand. In contrast, the PDF magazine *OxConnect* lies within this zone. The average focus score was also highest for *OxConnect* and lowest for *Hybrid Magazine*. When examining the scores for focus (Figure 1A) and cognitive demand (Figure 1B), we found that the *Hybrid Magazine*’s focus score (mean = 49.34619) was neither too high nor too low, indicating a balance between the number of items competing for attention and the few that draw the most attention. This balance suggests that *Hybrid Magazine* is likely not to overwhelm its readers with content but is also not too easy to process, which could result in maintaining its audience’s viewing time. On the other hand, *The Oxford Student* magazine had a lower score for cognitive demand (mean = 74.15509), which is similar to the score for focus (mean = 60.01919). After considering the frame-by-frame attention trends, which are discussed in further detail in the following section, it becomes clear that *The Oxford Student* magazine is not as engaging as the other two magazines. The results of our analysis show that the focus for *OxConnect* magazine was found to be the highest (mean = 66.08838), indicating that although some articles may receive much attention, they are relatively few. Additionally, *OxConnect*’s cognitive demand score (mean = 77.21173) fell within the high zone, which may overwhelm some readers while offering positively engaging little. This led us to investigate the relationship between the three magazines’ cognitive demand and focus scores to see if their high cognitive demand scores could be positively channelled with a high user focus. *OxConnect*‘s Pearson’s correlation = 0.01565399, t(46,306) = 3.369, and *p* = 0.0007551, but both *Hybrid Magazine* (Pearson’s correlation = 0.4084605, t(1566) = 13.208, *p* < 0.001) and *The Oxford Student* (Pearson’s correlation = 0.3618397, t(1158) = 17.708, *p* < 0.001) showed a moderate positive correlation (see Figure 2), suggesting that their high cognitive demand scores may not be high enough to overwhelm readers but are optimal sufficient to be correlated with high focus. However, with *OxConnect* having a very weak correlation between focus and cognitive demand, it may cancel out the overall positive effect of high focus, as its cognitive demand score falls in the high zone and has a negligible relationship with high focus scores. Online magazines generally demonstrated a more balanced range of these metrics than PDF magazines. Although the online environment and users’ familiarity with the web may provide some justification for this conclusion, it is also supported by prior research [4] that magazines may also transition from the web to PDFs, given the increasing popularity of online magazines [57], as evidenced by consumers’ inclination to favour modes that align with their established habits and routines [58]. The significance of examining these measures together becomes evident when their interrelationships are considered (see Figure 3). Assessing them individually may lead to misleading results. Although high cognitive demand in specific sections could enhance focus [19], it is crucial to contextualise the data in the media format and other qualitative and quantitative metrics to gauge the overall impact on engagement. A comprehensive analysis of all the trends is necessary to avoid confusion.

### 4.2. User Attention

**Figure 4:**
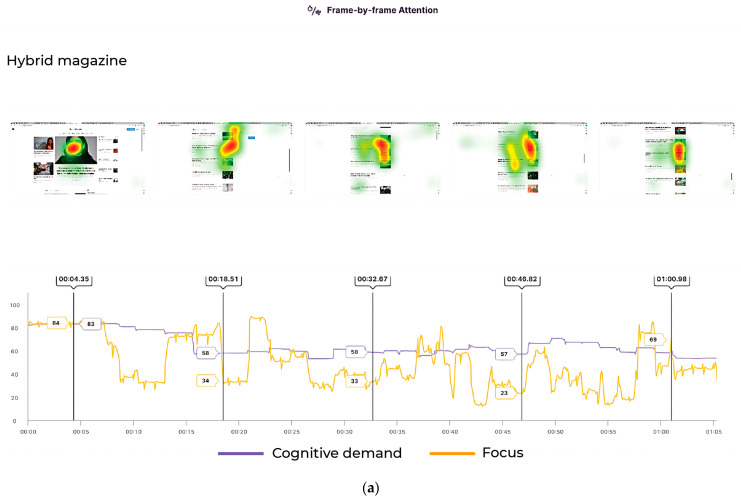

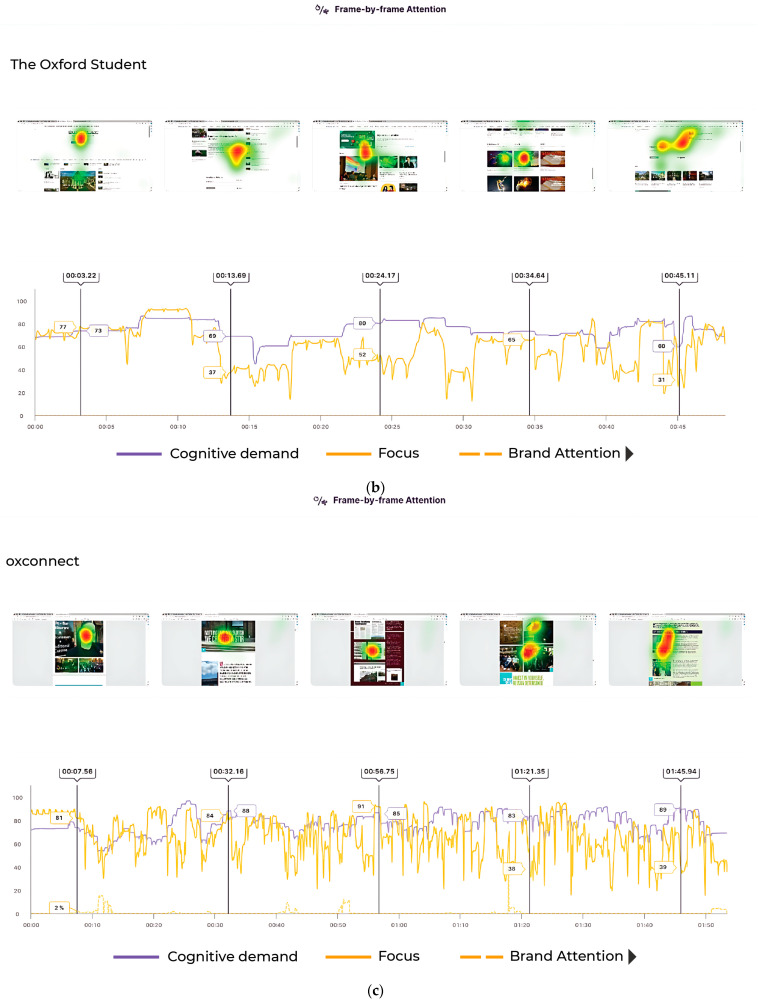
(**a**) Heat-map data for some of the frames for *Hybrid Magazine*. (**b**) Heat-map data for some of the frames for *The Oxford Student*. (**c**) Heat-map data for some of the frames for *OxConnect*.

**Figure 5:**
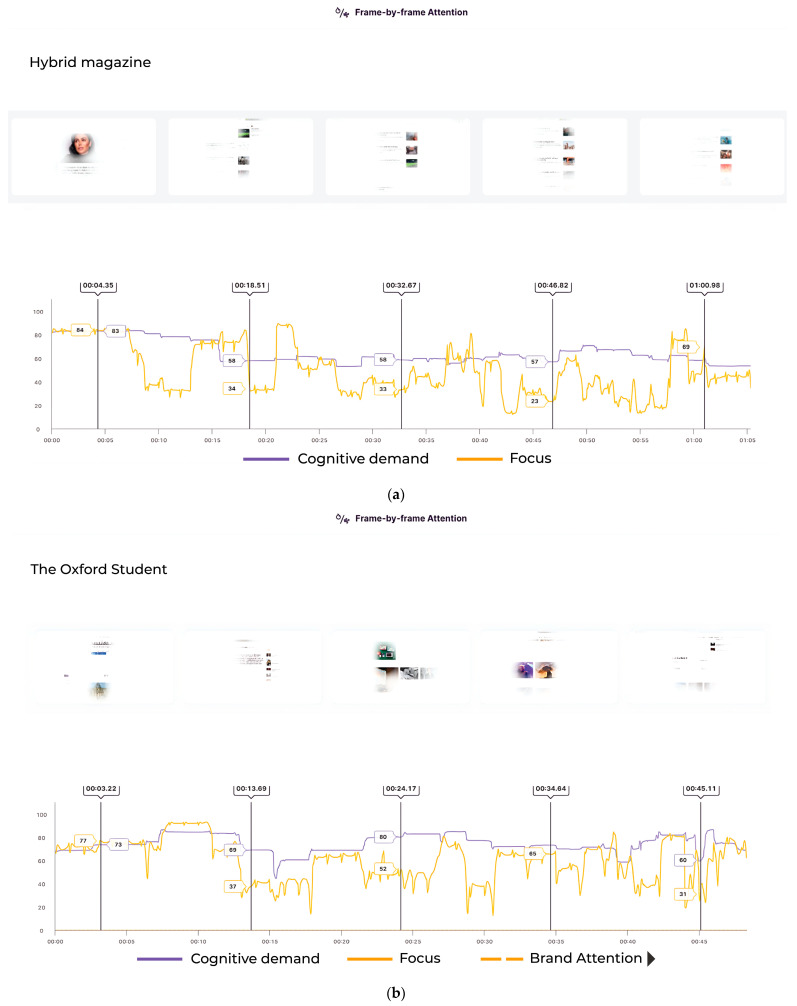

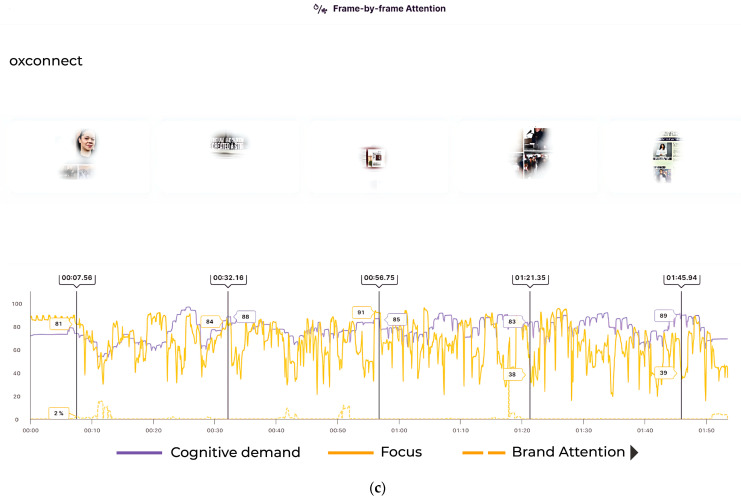
(**a**) Fog-map data for some of the frames for *Hybrid Magazine*. (**b**) Fog-map data for some of the frames for *The Oxford Student*. (**c**) Fog-map data for some of the frames for *OxConnect*.

One of our objectives was to evaluate these magazines regarding user engagement, where we identified attention heat maps (see Figure 4a–c) and fog maps (see Figure 5a–c) for each magazine. Attention heat maps show how customers look at their assets by highlighting the areas that are most likely to catch people’s eyes when they see the video. Colours ranging from green to yellow to red indicate the cumulative time of eye fixations to each region of an image or video. Warmer colours indicate greater attention. Recurring patterns emerged in the heat maps for all three magazines, demonstrating that the readers were particularly drawn to visually appealing information. The Attention Fog Map is grounded in the same projected attention as the Attention Heat Map and is often called a “reverse heatmap.” Instead of applying colour to areas where attention is present, it covers the entire image or video in white fog, which clears to reveal areas with significant visual attention. If something is not visible on the Attention Fog Map, it is unlikely to be seen by readers. This model is grounded in a unique floating window operation that spans a 2 s, ensuring precise, human-like attention behaviour predictions. By constantly analysing the shifting visual landscape of a video, this model can highlight those moments and elements most likely to seize a viewer’s gaze.

**Figure 6:**
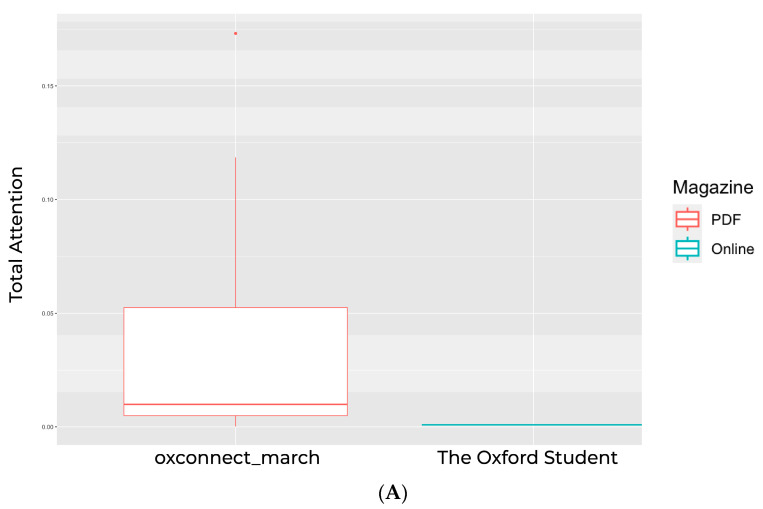

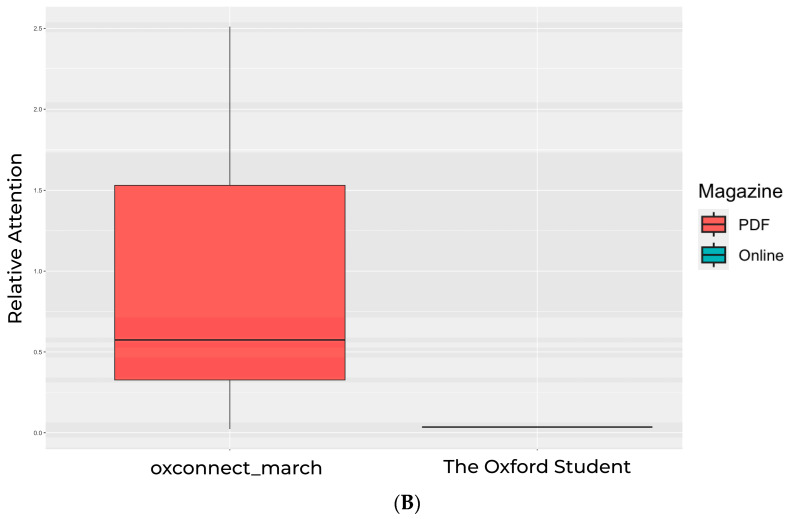
(**A**) The difference in total attention between online and PDF magazines. (**B**) Difference in relative attention between online and PDF magazines (after excluding the high-influence outliers).

For *Hybrid Magazine*, attention was primarily concentrated on the face, which occupied most of the space in the first frame and served as a salient feature that captured great attention. The pattern continued, indicating that readers were likely to shift their focus to visuals in the top half of the frame, a trend observable across all frames. Moreover, the data suggest that readers connect visual information with the adjacent textual information. However, when comparing *The Oxford Student* magazine to the *Hybrid Magazine*, the difference in reader attention was noticeable in the focus location. Unlike *Hybrid Magazine*, readers of *The Oxford Student* magazine were found to pay more attention to the centre of the frames. This difference could be due to the layout of the two magazines and how they affect the elements that drive the reader’s attention. Additionally, more attention was paid to the textual content in *The Oxford Student* magazine, possibly because it was displayed in a more spread-out manner, which did not make readers feel overstimulated. It is worth noting that *OxConnect* magazine had a significant amount of textual content, but little attention was paid to it. This further validated the focus and cognitive demand scores that suggested insufficient areas for readers to attend to. The difference in how the text was positioned in the *OxConnect* magazine compared to *The Oxford Student* magazine indicated that when content feels too text-heavy, it may result in a more rigid structure with little scope for dynamism, thereby driving readers’ attention away from it. This emphasises the importance of content layout and dimensions, as other studies also supported [59]. While presenting the information in reverse order, the fog map data unveiled the previously contrasted aspects. For *Hybrid Magazine*, the trend pointed towards a high level of focus, where the textual and visual content was placed nearby for easy referencing and increased engagement, thereby making the content structure feel more customised to the user’s needs. This personalisation leads to greater individualised interaction with the content, boosting user engagement [60] and supporting our hypothesis. In several instances, while publishing *OxConnect* magazine, it was observed that when cognitive demand was high, focus increased. This suggests it captured readers’ attention even when the content was cognitively demanding. This trend showed the potential for simplicity in the layout of visually engaging yet demanding content throughout the magazine. When analysing the fluctuating data for *OxConnect*, it was observed that the text-heavy presentation of the content may have overwhelmed readers and disrupted the flow of focus, which could have been more consistent. Additionally, a disparity in the colour scheme for this magazine was noticed, which may have caused inconsistencies and increased task-switching costs for readers, hindering connectivity within the magazine and potentially affecting user interactions. Placing large sections of content on dark-coloured backgrounds may also disrupt the aesthetic required for user engagement and personalisation, which is a highly influential factor in readers’ intent to engage with the content [25]. The software generated attention scores only for video frames that featured automatic areas of interest (AOIs) detected through convolutional neural networks (CNNs) and advanced deep-learning techniques. We were not able to select the AOIs manually in the video. This option is available only for images and not videos. The selected AOIs on all three magazines were matched and detected using the Predict algorithm, built on a multilayer perceptron that taps into continuously updated state-of-the-art object-detection models. The algorithm was trained on extensive datasets comprising thousands of distinct logos and object instances and built on the Google Logo database. If a logo is not listed in the database, the algorithm will not recognise it as an AOI or mark it on the video. The software detected no automatic AOIs in *Hybrid Magazine*, 1 in *The Oxford Student* (“Scitech”), and 17 in *OxConnect*. Consequently, the relative attention scores (brand attention relative to other logos is bottom-up attention, which is automatic happens in human behaviour. Our software measures and predicts what people look at in the first 2 s and total attention scores (areas attracting consumers throughout the exposure). “Start Attention” (focal points for consumers during the initial 2 s of a 5-second exposure) and “End Attention” (focal points for consumers during the concluding 2 s of a 5-second exposure), with low-attention areas implying lesser importance for consumers and high-attention areas being important to sustain and enhance for maintaining consumers’ interests, were higher for the PDF magazine than for the online magazine. However, this finding must be approached with caution because of the highly skewed scores caused by the lack of AOI data for online magazines and the significant difference in AOIs detected between online and PDF magazines (as shown in Figure 6A,B). Despite our desire to investigate this aspect, we compared the means of the available data (excluding any high-influence outliers; see Appendix G for the difference in relative attention between online and PDF magazines without removing outliers). Our findings revealed that “Scitech” received minimal attention in *The Oxford Student* magazine, with a mean total attention of 0.0009170334 and a mean relative attention of 0.03545862, which differed statistically significantly from the total attention (t(16) = 2.9115, *p* < 0.05) of *OxConnect* (mean total attention = 0.03656817) and from the relative attention (t(16) = 2.8143, *p* < 0.05) of *OxConnect* (mean relative attention = 0.9338123) (refer to Table 5). In terms of availability (measuring the duration in seconds that a brand is present in a video, which relates to the “frame-by-frame attention”), despite the variation in the AOI data, the duration of each brand detected did not differ significantly (t(16) = 1.8295, *p* = 0.08602) between online (mean = 1.25) and PDF (mean = 2.294118) magazines.

In addition to the data disparity, it seemed logical to investigate the differences in AOIs within the *OxConnect* magazine. Therefore, we analysed the AOIs with the highest and lowest attention and availability scores and found that “Oxford Instruments” scored the highest for total attention (0.1731328857) and availability (10.000). Upon verifying this score with the video frames, the only noteworthy pattern that emerged was related to the visuals and their appeal for this AOI. However, after exploring the AOIs, particularly those with the lowest attention and availability scores, no other significant trends were observed across the video frames for the *OxConnect* magazine. Nonetheless, this analysis highlighted the need for online magazines to pay more attention to their brand, as this could enhance the overall user experience for their publications.

## 5. Discussion

After analysing the available data and trends, we identified several practices that could benefit magazines. First, there is a growing preference for online magazines, which may not seem like a significant change, but it can affect overall user engagement. Therefore, magazine publishers may consider transitioning from PDF magazines to websites, which can provide a more consistent experience and more straightforward navigation. This approach might also be beneficial because users are more accustomed to using apps and websites, and it offers more freedom in separating sections in which readers may be interested. This could help readers freely explore their sections of interest in the order they prefer, rather than constantly scrolling between sections, which may disrupt their reading flow and lead to a loss of interest. This finding aligns with prior research that suggests users prefer paging over scrolling [61].

Second, there is a growing emphasis on the significance of high-quality visual content, which has become a key trend. Choosing the correct visual content is essential, but this alone may not be sufficient. Other factors, such as the layout and positioning of the content, are equally important. Research has shown that readers link information when exposed to stimuli, so it is vital to place visual and textual content within easy reach of each other. This can be achieved through a balanced layout and visual and textual content interspersing. The interaction between visual and textual content can lead to greater personalisation and dynamic engagement for readers, making the content more appealing and engaging. Visual aids also enable smooth navigation, making it easier for readers to find what they want. However, a lack of balance can result in a constrained screen space with little room for choice and decision-making, potentially affecting readers’ purchasing behaviours. Therefore, it is vital to redesign with a focus on simplicity and clarity, incorporating only necessary and sufficient content. This approach can help create a more engaging and practical visual experience for readers [62,63].

Third, it is essential to consider colour choices concerning text fonts and the amount of textual content, as they can elicit affective responses in readers [64], and large blocks of text with small inter-line spaces and smaller fonts can be overwhelming, particularly when placed on dark-coloured backgrounds that are inconsistent. Therefore, it is necessary to maintain consistency in colour choices and use a palette that helps users connect with the brand’s values for brand recall and optimal information processing and preference [65]. The same applies to text fonts, which should be consistent with the chosen colours and the recommendation mentioned above for balance in generating positive responses in terms of valence.

**Figure 7:**
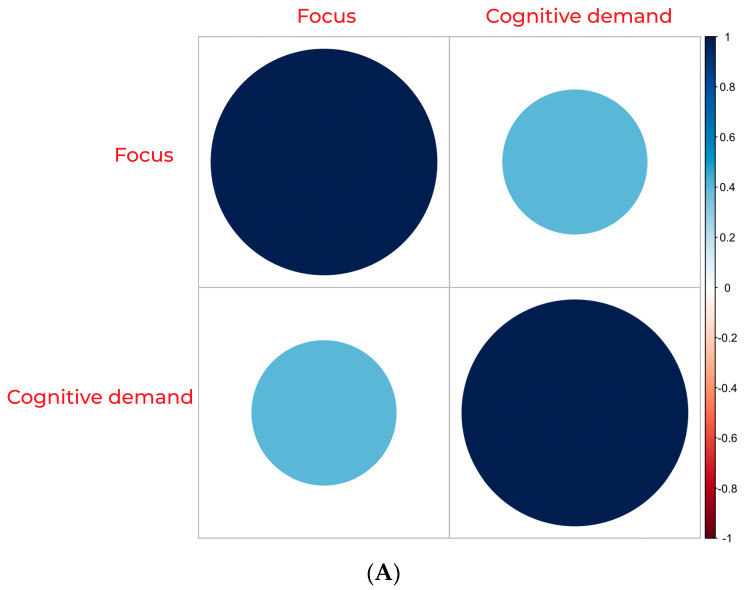

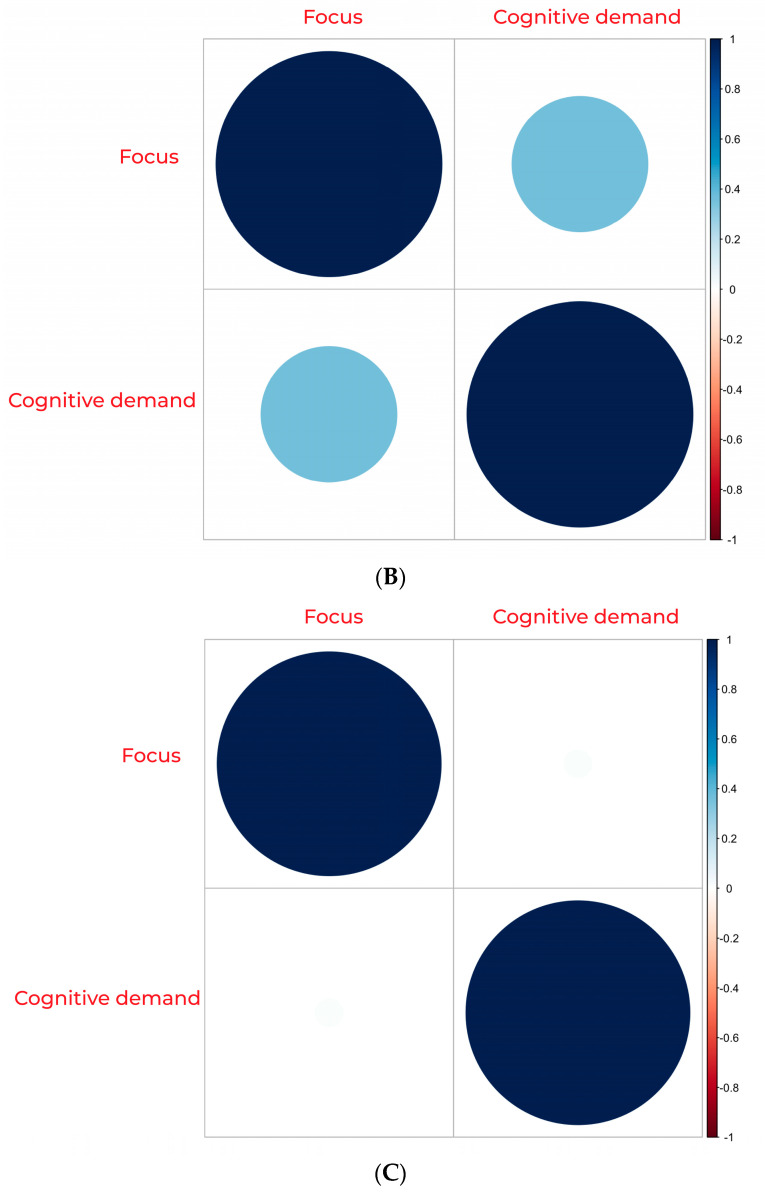
(**A**) Correlation between focus and cognitive demand for *Hybrid Magazine*. (**B**) Correlation between focus and cognitive demand for *The Oxford Student*. (**C**) Correlation between focus and cognitive demand for *OxConnect*.

It is crucial to consider the motivation behind the medium and the factors that affect it when making changes. It is essential to note user trends because the data indicate that online magazines, such as *Hybrid Magazine* (see Figure 7A) and *The Oxford Student* (see Figure 7B), have a moderate correlation between cognitive demand and focus, unlike the PDF magazine *OxConnect* (see Figure 7C). Therefore, the structure of the textual and visual content should be designed to reduce cognitive load, which is unnecessary, and make it more engaging where necessary by considering the recommendations. Additionally, the findings suggest that capturing user attention through logos and brands can provide valuable insights into the overall attention and availability scores and that a lack of brand emphasis can lead to a lack of potentially vital data that can be used to improve magazines. Therefore, online magazines should focus on brand emphasis at critical points across their magazines to enhance their attention and availability scores, while also making the AOIs more engaging.

### Limitations of Study

While this research aimed to identify trends in user behaviour and experiences across various magazines, certain aspects require careful interpretation. As previously discussed, examining the data in isolation may lead to misleading conclusions. Therefore, a comprehensive understanding of all the variables is crucial. For instance, it may seem that one magazine has a higher level of focus than another. Still, it is difficult to determine the underlying reasons and potential implications without considering cognitive demands and other factors, such as heat maps and fog maps. Additionally, some measures are unavailable due to limited brand declarations in the referenced databases, which must be considered when comparing data. Furthermore, since this study employed a novel approach using neuromarketing AI eye-tracking research prediction software to generate data, there are inherent limitations, such as missing data for specific measures. Therefore, it is essential to maintain margins of error when using these methods, especially considering the growing popularity of AI-based prediction software in commercial settings. The ever-increasing popularity of AI eye-tracking-based prediction software in commercial settings is evident in various research studies. These technologies offer a wide range of applications, from predicting human movement intentions [66] to analysing viewer reactions to images [67] and improving VR applications through path predictions based on eye movements [31]. While neuromarketing human behaviour prediction AI-based software is more cost-effective and time-efficient than researching live participants, it requires extensive knowledge of the neuromarketing discipline to interpret data effectively. This is because the software provides all research data in one Excel sheet, which may be overwhelming for non-experts. In addition, a high level of statistical expertise is required to obtain precise outcomes.

## 6. Conclusions

A new modern step in reading-behaviour research involves utilising neuromarketing eye-tracking AI prediction software to unravel the complexities of visual attention [31,68,69]. This innovative approach combines the benefits of eye-tracking technology with artificial intelligence models to accurately predict consumer behaviour and preferences [67]. In conclusion, this study represents a new modern step in reading-behaviour research to unravel the complexities of visual attention by employing neuromarketing eye-tracking AI prediction software, providing valuable insights into the advantages and disadvantages of this technology concerning online and PDF college magazines. By cultivating a nuanced understanding of user behaviour, this study aims to facilitate well-informed design decisions that enhance the overall reading experience and maximise the impact of college magazines in the digital age.

With this research, we proved how modern eye-tracking AI technologies can be employed in the neuromarketing discipline to gain more information about various types of attention, such as focus, engagement, cognitive demand, and clarity, which we measured in depth with our study. By comparing eye-tracking results with AI predictions, researchers can gain valuable insights into how individuals interact with visual stimuli, aiding scientific research and real business applications [70]. The benefits of this approach are that it may be worthwhile to delve further into automated datasets, which could potentially improve their reliability. Because the exploration of such methods is still in its early stages, future research should make them more accessible by validating their performance using additional metrics. To gain richer insights from the results and provide a new direction in the field of neuromarketing, we need to compare these metrics with existing benchmarks.

This research also lays the groundwork for future studies to examine user engagement versus user involvement in greater detail, considering additional nuances that can enhance critical factors, such as brand recall, purchasing behaviour, brand perception, and lasting impressions. AI-based applications such as Visual Attention Software and Predict enhance visual attention-pattern analyses and provide high accuracy with detailed fixation-point probability maps and sequence estimations. Integrating neuromarketing, eye-tracking technology, and AI software has significantly advanced the understanding of consumer intent and optimised marketing strategies.

## 7. Implications

AI-driven eye-tracking technology has significant implications for predicting human behaviour, enhancing various applications from collaborative robotics to user interface design. Collaborative robots, for instance, can benefit from AI systems that predict human arm movement based on eye gaze, allowing robots to adapt their actions in real time and improve interaction efficiency and safety in industrial settings [66,71]. The literature suggests that while there is a growing body of research on the application of artificial intelligence (AI) in understanding and predicting consumer behaviour, specific exploration into the use of AI with eye-tracking technology for this purpose appears to be less developed. Studies have acknowledged the potential of AI in marketing and consumer-behaviour analysis, with some focusing on integrating AI and eye tracking [35,72]. However, the literature indicates a need for more focused research on the synergistic use of these technologies to predict consumer behaviour. This research has significantly contributed to this field, as it genuinely represents how AI eye-tracking consumer-behaviour software can be successfully used in neuromarketing disciplines. The software utilised in our study was highly accurate, as it was developed in collaboration with Stanford University and incorporates cutting-edge scientific and machine-learning techniques. The accuracy and efficiency of these systems have been demonstrated through experiments utilising eye-tracking devices such as the Tobii X120 [73] and innovative solutions such as the EyeDee-embedded eye-tracking system [74]. These advancements showcase the potential for AI-driven eye-tracking software to revolutionise industries such as gaming, education, marketing, and more by providing valuable insights and enhancing user experiences through predictive capabilities [75,76,77,78]. Given that this paper presents pioneering research in demonstrating how neuromarketing AI-based consumer behaviour-prediction software can provide substantial value in measuring, comprehending, predicting, and influencing consumer behaviour, it is imperative to continuously update the metrics used to assess the algorithm’s upgraded versions, including their operational definitions and benchmarks, after accounting for their regressors and the factors that must be controlled for. The future recommendation would be to broadly develop sensors such as emotions, memory, cognition, and attention, as all of these metrics significantly affect behaviour. Predicting memory-recall learning engagement would be more straightforward, which drives consumer preferences and decisions. AI models trained on eye-movement data can accurately predict emotional intelligence (EI) profiles, revealing how individuals with different EI profiles respond to visual stimuli [79]. Owing to the constraints of the AI eye-tracking prediction software (Predict), we could not download our research video with the option of manually pausing the video by selecting the high vs. lower peaks of focus and cognitive demand in each time frame. It is highly advisable to develop this option and not rely on the options the software developer preselected. Furthermore, the software calculates only two metrics (focus and cognitive demand) for video format testing. If we want to obtain more metrics (such as the software having the option for image testing, where there are added clarity and engagement metrics), it would be great to consider adding this option in the future. The software is well-designed and user-friendly, but if it were possible to download all 180,000 recordings while testing as a single column each, it would be much easier to perform various statistical calculations instead of obtaining all the results from the research in one Excel column for each metric. This predictive capability is rooted in the understanding that eye movements are anticipatory of task steps, making them reliable indicators of human intentions [37]. Integrating AI and deep learning techniques in analysing eye-tracking data has also captured a significant portion of the explainable variance in human eye movement patterns, suggesting that these models can support spatial-attention and object-recognition tasks [80]. In summary, AI eye-tracking consumer-behaviour software presents a significant opportunity for advancing marketing strategies by providing a deeper understanding of consumer engagement. The integration of eye tracking with AI has been shown to enhance marketing communication, personalise consumer experiences, and potentially reduce cognitive workload [81]. Unfortunately, there is no evidence of the gender correlations from the total sample size the algorithm was built on, nor are there specifications for the geographical locations where this recording from the largest neuromarketing database was taken. While promising, the responsible and ethical use of these technologies is imperative to address potential challenges and ensure consumer trust [72,82].

## Figures and Tables

**Table 1:** Welch’s Two-Sample *t*-tests between every pair of magazines for cognitive demand.

Estimates	Estimate 1	Estimate 2	Statistic	*p*-Value	Parameter	Conf. Low	Conf. High	Method	Alternative
1: *Hybrid Magazine* 2: *The Oxford Student*	64.70426	74.15509	−29.019	<0.001	2722.9	−10.089445	−8.812227	Welch’s Two-Sample *t*-test	two-sided
1: *Hybrid Magazine* 2: *OxConnect*	64.70426	77.21173	−50.705	<0.001	1655.5	−12.99129	−12.02365	Welch’s Two-Sample *t*-test	two-sided
1: *OxConnect* 2: *The Oxford Student*	77.21173	74.15509	13.876	<0.001	13.876	2.624477	3.488791	Welch’s Two-Sample *t*-test	two-sided

**Table 2:** One-way ANOVA test for all 3 magazines for cognitive demand.

	Df	Sum Sq	Mean Sq	F Value	Pr (<F)
Magazines	2	245,195	122,597	1605	<2 × 10^−16^ ***
Residuals	49,033	3,746,212	76		

*** Indicates a high significance (*p* < 0.001).

**Table 3:** Welch’s Two-Sample *t*-tests between every pair of magazines for focus.

Estimates	Estimate 1	Estimate 2	Statistic	*p*-Value	Parameter	Conf. Low	Conf. High	Method	Alternative
1: *Hybrid Magazine* 2: *The Oxford Student*	49.34619	60.01919	−14.68	<0.001	2694.5	−12.0986	−9.24742	Welch’s Two-Sample *t*-test	two-sided
1: *Hybrid Magazine* 2: *OxConnect*	49.34619	66.08838	−31.55	<0.001	1635.9	−17.783	−15.7014	Welch’s Two-Sample *t*-test	two-sided
1: *OxConnect* 2: *The Oxford Student*	66.08838	60.01919	11.927	<0.001	1214.5	5.070804	7.067574	Welch’s Two-Sample *t*-test	two-sided

**Table 4:** One-way ANOVA test for all 3 magazines for focus.

	Df	Sum Sq	Mean Sq	F Value	Pr (<F)
Magazines	2	459,638	229,819	812.7	<2 × 10^−16^ ***
Residuals	49,033	13,866,203	283		

*** Indicates a high significance (*p* < 0.001).

**Table 5:** Welch’s Two-Sample *t*-tests between online and PDF magazines for total attention, relative attention, and availability.

Measure	Online	PDF	Statistic	*p*-Value	Parameter	Conf. Low	Conf. High	Method	Alternative
Total attention	0.000917	0.036568	2.9115	<0.05	16	0.01061	0.062526	Welch’s Two-Sample *t*-test	two-sided
Relative attention	0.035459	0.933812	4.4552	<0.001	14	0.50133	1.366294	Welch’s Two-Sample *t*-test	two-sided
Availability	1.25	2.294118	1.8295	0.08602	16	1.084275	3.503961	Welch’s Two-Sample *t*-test	two-sided

**Figure A1:**
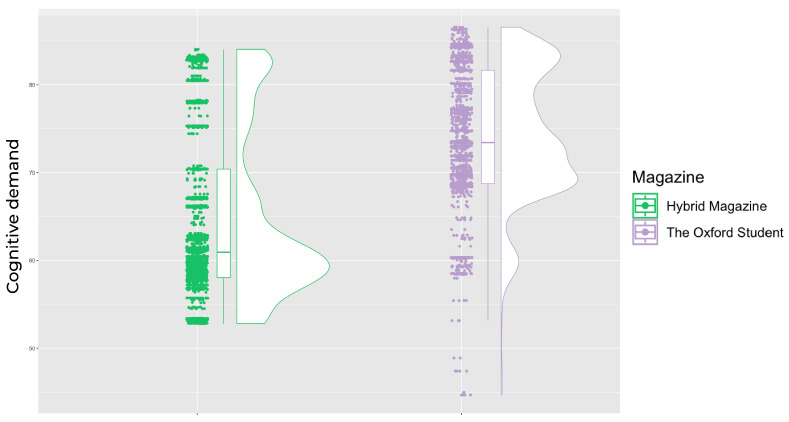
Difference in cognitive demand between *Hybrid Magazine* and *The Oxford Student*.

**Figure A2:**
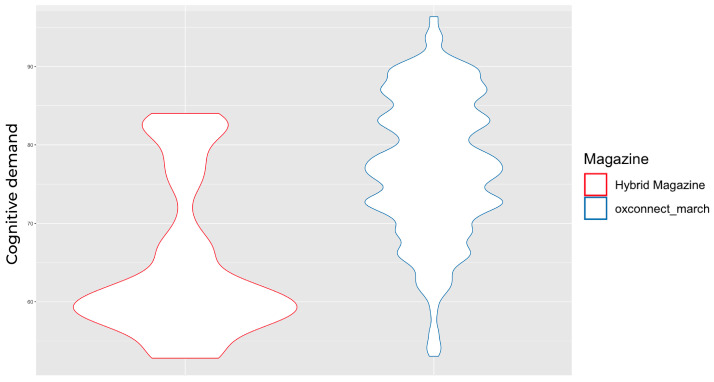
The difference in cognitive demand between *Hybrid Magazine* and *OxConnect*.

**Figure A3:**
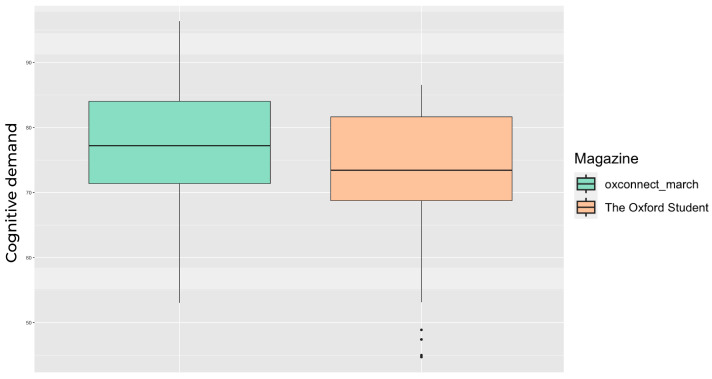
The difference in cognitive demand between *OxConnect* and *Oxford Student*.

**Figure A4:**
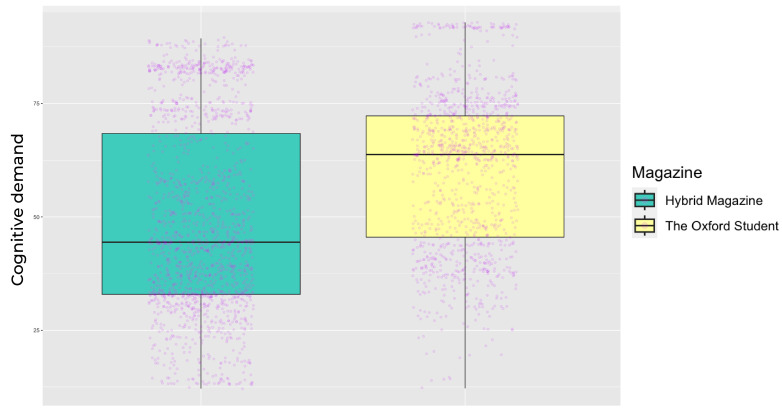
Difference in focus between *Hybrid Magazine* and *The Oxford Student*.

**Figure A5:**
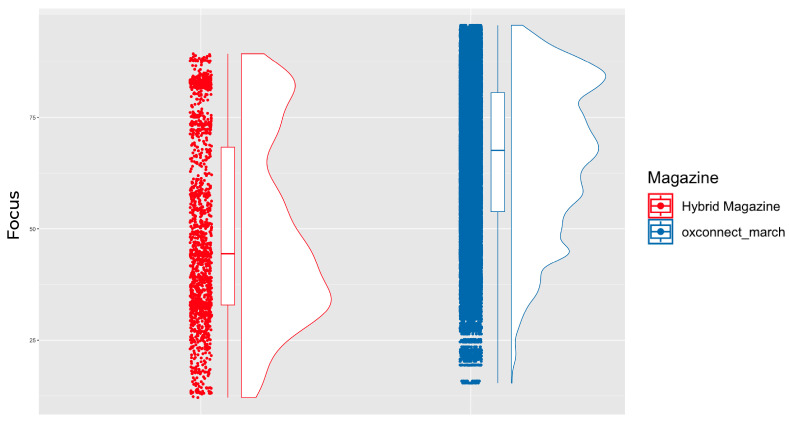
Difference in focus between *Hybrid Magazine* and *OxConnect*.

**Figure A6:**
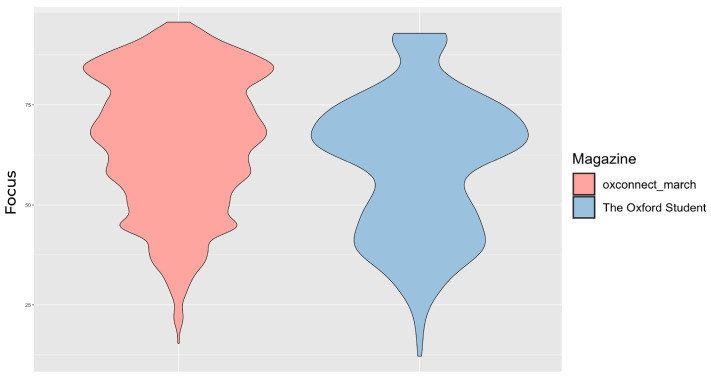
Difference in focus between *OxConnect* and *The Oxford Student* magazines.

**Figure A7:**
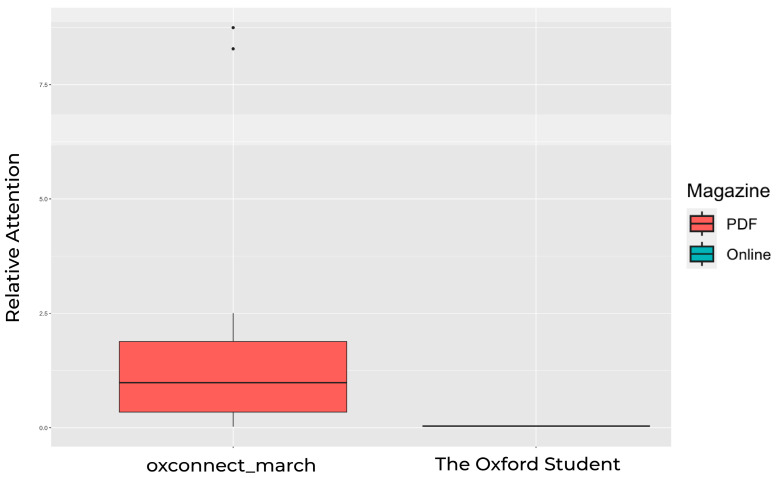
Difference in relative attention between online and PDF magazines (including outliers).

**Figure A8:**
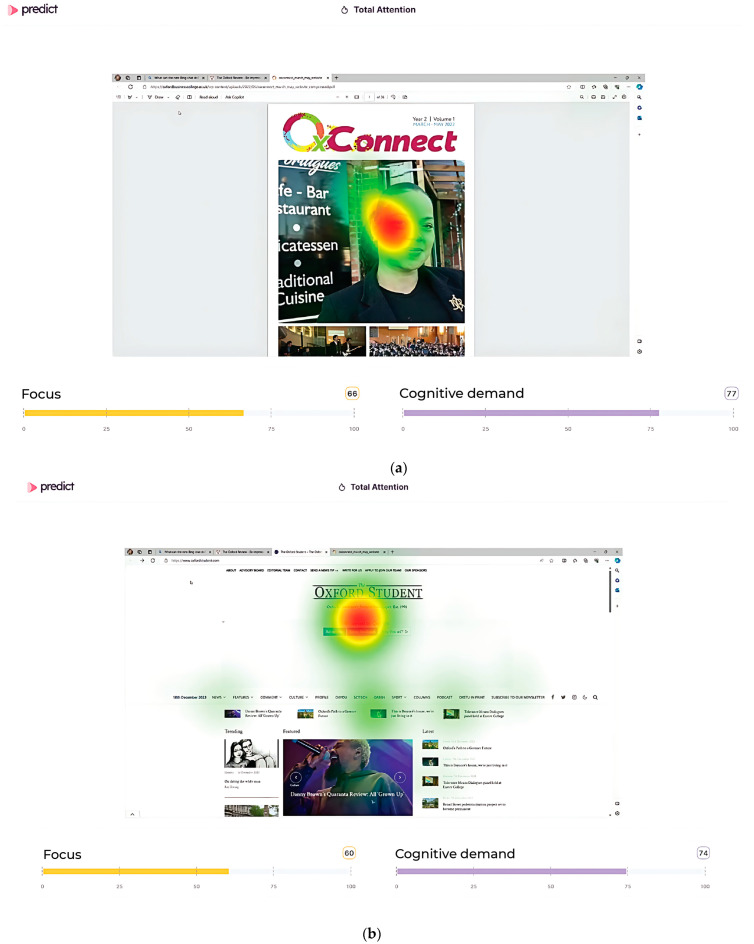

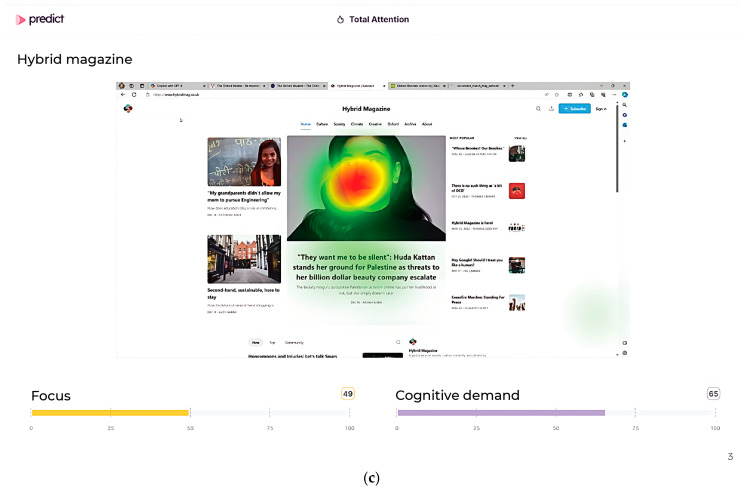
(**a**) Neuromarketing results for *OxConnect*’s main page; (**b**) Neuromarketing results for *The Oxford Student*’s main page; (**c**) Neuromarketing results for *Hybrid Magazine*’s main page.

## Data Availability

The data supporting this study’s findings are available in Figshare at DOI:10.6084/m9.figshare.26180921. These data were published under CC BY 4.0. Deed Attribution 4.0. International license.

## Abbreviations

AI	artificial intelligence
CNNs	convolutional neural networks
DNN	deep neural networks
MMI	multimedia information
Predict	consumer-behaviour AI eye-tracking prediction software
ET	eye tracking
CD	cognitive demand
AOI	area of interest
EI	emotional intelligence

## References

[1] King A.J., Bol N., Cummins R.G., John K.K. (2019). Improving Visual Behavior Research in Communication Science: An Overview, Review, and Reporting Recommendations for Using Eye-Tracking Methods. Commun. Methods Meas..

[2] Brunyé T.T., Drew T., Weaver D.L., Elmore J.G. (2019). A review of eye tracking for understanding and improving diagnostic interpretation. Cogn. Res. Princ. Implic..

[3] Latini N., Bråten I., Salmerón L. (2020). Does reading medium affect processing and integration of textual and pictorial information? A multimedia eye-tracking study. Contemp. Educ. Psychol..

[4] Wang P., Chiu D.K., Ho K.K., Lo P. (2016). Why read it on your mobile device? Change in reading habit of electronic magazines for university students. J. Acad. Librariansh..

[5] Wu M.-J., Zhao K., Fils-Aime F. (2022). Response rates of online surveys in published research: A meta-analysis. Comput. Hum. Behav. Rep..

[6] Bonner E., Roberts C. (2017). Millennials and the Future of Magazines: How the Generation of Digital Natives Will Determine Whether Print Magazines Survive. J. Mag. Media.

[7] Barral O., Lallé S., Guz G., Iranpour A., Conati C. (2020). Eye-Tracking to Predict User Cognitive Abilities and Performance for User-Adaptive Narrative Visualizations. Proceedings of the 2020 International Conference on Multimodal Interaction.

[8] Klaib A.F., Alsrehin N.O., Melhem W.Y., Bashtawi H.O., Magableh A.A. (2021). Eye tracking algorithms, techniques, tools, and applications with an emphasis on machine learning and Internet of Things technologies. Expert Syst. Appl..

[9] Sharma K., Giannakos M., Dillenbourg P. (2020). Eye-tracking and artificial intelligence to enhance motivation and learning. Smart Learn. Environ..

[10] Yi T., Chang M., Hong S., Lee J.-H. (2021). Use of Eye-tracking in Artworks to Understand Information Needs of Visitors. Int. J. Hum. Comput. Interact..

[11] Novák J., Masner J., Benda P., Šimek P., Merunka V. (2023). Eye Tracking, Usability, and User Experience: A Systematic Review. Int. J. Hum. Comput. Interact..

[12] Vehlen A., Spenthof I., Tönsing D., Heinrichs M., Domes G. (2021). Evaluation of an eye tracking setup for studying visual attention in face-to-face conversations. Sci. Rep..

[13] Gerstenberg T., Peterson M.F., Goodman N.D., Lagnado D.A., Tenenbaum J.B. (2017). Eye-Tracking Causality. Psychol. Sci..

[14] Olan F., Suklan J., Arakpogun E.O., Robson A. (2021). Advancing Consumer Behavior: The Role of Artificial Intelligence Technologies and Knowledge Sharing. IEEE Trans. Eng. Manag..

[15] Chaudhary K., Alam M., Al-Rakhami M.S., Gumaei A. (2021). Machine learning-based mathematical modelling for prediction of social media consumer behavior using big data analytics. J. Big Data.

[16] Nunes C.A., Ribeiro M.N., de Carvalho T.C., Ferreira D.D., de Oliveira L.L., Pinheiro A.C. (2023). Artificial intelligence in sensory and consumer studies of food products. Curr. Opin. Food Sci..

[17] Al Adwan A., Aladwan R. (2022). Use of artificial intelligence system to predict consumers’ behaviors. Int. J. Data Netw. Sci..

[18] Hakami N.A., Mahmoud H.A.H. (2022). The Prediction of Consumer Behavior from Social Media Activities. Behav. Sci..

[19] Gkikas D.C., Theodoridis P.K., Beligiannis G.N. (2022). Enhanced Marketing Decision Making for Consumer Behaviour Classification Using Binary Decision Trees and a Genetic Algorithm Wrapper. Informatics.

[20] Pop Ș., Pelau C., Ciofu I., Kondort G. (2023). Factors Predicting Consumer-AI Interactions. Proceedings of the 9th BASIQ International Conference on New Trends in Sustainable Business and Consumption.

[21] Kar A. (2020). MLGaze: Machine Learning-Based Analysis of Gaze Error Patterns in Consumer Eye Tracking Systems. Vision.

[22] Li Y., Zhong Z., Zhang F., Zhao X. (2022). Artificial Intelligence-Based Human–Computer Interaction Technology Applied in Consumer Behavior Analysis and Experiential Education. Front. Psychol..

[23] Margariti K., Hatzithomas L., Boutsouki C. (2023). Implementing Eye Tracking Technology in Experimental Design Studies in Food and Beverage Advertising. Consumer Research Methods in Food Science.

[24] Pfeiffer J., Pfeiffer T., Meißner M., Weiß E. (2020). Eye-Tracking-Based Classification of Information Search Behavior Using Machine Learning: Evidence from Experiments in Physical Shops and Virtual Reality Shopping Environments. Inf. Syst. Res..

[25] Deng R., Gao Y. (2023). A review of eye tracking research on video-based learning. Educ. Inf. Technol..

[26] Lim J.Z., Mountstephens J., Teo J. (2022). Eye-Tracking Feature Extraction for Biometric Machine Learning. Front. Neurorobot..

[27] Houpt J.W., Frame M.E., Blaha L.M. (2018). Unsupervised parsing of gaze data with a beta-process vector auto-regressive hidden Markov model. Behav. Res. Methods.

[28] Darapaneni N., Prakash M.D., Sau B., Madineni M., Jangwan R., Paduri A.R., Jairajan K.P., Belsare M., Madhavankutty P. (2022). Eye Tracking Analysis Using Convolutional Neural Network. Proceedings of the 2022 Interdisciplinary Research in Technology and Management (IRTM).

[29] Yin Y., Juan C., Chakraborty J., McGuire M.P. Classification of Eye Tracking Data using a Convolutional Neural Network. Proceedings of the 17th IEEE International Conference on Machine Learning and Applications.

[30] Yin Y., Alqahtani Y., Feng J.H., Chakraborty J., McGuire M.P. (2021). Classification of Eye Tracking Data in Visual Information Processing Tasks Using Convolutional Neural Networks and Feature Engineering. SN Comput. Sci..

[31] Ahtik J. (2023). Using artificial intelligence for predictive eye-tracking analysis to evaluate photographs. J. Graph. Eng. Des..

[32] Wu A.X., Taneja H., Boyd D., Donato P., Hindman M., Napoli P., Webster J. (2020). Computational social science: On measurement. Science.

[33] Roy A., Saffar M., Vaswani A., Grangier D. (2021). Efficient Content-Based Sparse Attention with Routing Transformers. Trans. Assoc. Comput. Linguist..

[34] Colin R., Minh-Thang L., Peter J.L., Ron J.W., Douglas E. Online and linear-time attention by enforcing monotonic alignments. Proceedings of the 34th International Conference on Machine Learning—Volume 70.

[35] Zhang Z., Ma J., Du J., Wang L., Zhang J. (2023). Multimodal Pre-Training Based on Graph Attention Network for Document Understanding. IEEE Trans. Multimed..

[36] Amin Z., Ali N.M., Smeaton A.F. (2021). Attention-Based Design and User Decisions on Information Sharing: A Thematic Literature Review. IEEE Access.

[37] Moe-Byrne T., Knapp P., Perry D., Achten J., Spoors L., Appelbe D., Roche J., Martin-Kerry J.M., Sheridan R., Higgins S. (2022). Does digital, multimedia information increase recruitment and retention in a children’s wrist fracture treatment trial, and what do people think of it? A randomised controlled Study Within A Trial (SWAT). BMJ Open.

[38] Gao Q., Li S. (2022). Impact of Online Courses on University Student Visual Attention During the COVID-19 Pandemic. Front. Psychiatry.

[39] Aily J.B., Copson J., Voinier D., Jakiela J., Hinman R., Grosch M., Noonan C., Armellini M., Schmitt L., White M. (2023). Understanding Recruitment Yield From Social Media Advertisements and Associated Costs of a Telehealth Randomized Controlled Trial: Descriptive Study. J. Med. Internet Res..

[40] Chen L., Jeong J., Simpkins B., Ferrara E. (2023). Exploring the Behavior of Users With Attention-Deficit/Hyperactivity Disorder on Twitter: Comparative Analysis of Tweet Content and User Interactions. J. Med. Internet Res..

[41] Mehmood A., Taber N., Bachani A.M., Gupta S., Paichadze N., A Hyder A. (2019). Paper Versus Digital Data Collection for Road Safety Risk Factors: Reliability Comparative Analysis From Three Cities in Low- and Middle-Income Countries. J. Med. Internet Res..

[42] Dorta-González P., Dorta-González M.I. (2023). The funding effect on citation and social attention: The UN Sustainable Development Goals (SDGs) as a case study. Online Inf. Rev..

[43] Guseman E.H., Jurewicz L., Whipps J. (2022). Physical Activity And Screen Time Patterns During The Covid-19 Pandemic: The Role Of School Format. Med. Sci. Sports Exerc..

[44] Byrne S.A., Reynolds A.P.F., Biliotti C., Bargagli-Stoffi F.J., Polonio L., Riccaboni M. (2023). Predicting choice behaviour in economic games using gaze data encoded as scanpath images. Sci. Rep..

[45] Vajs I.A., Kvascev G.S., Papic T.M., Jankovic M.M. (2023). Eye-Tracking Image Encoding: Autoencoders for the Crossing of Language Boundaries in Developmental Dyslexia Detection. IEEE Access.

[46] Liu L., Wang K.I.-K., Tian B., Abdulla W.H., Gao M., Jeon G. (2023). Human Behavior Recognition via Hierarchical Patches Descriptor and Approximate Locality-Constrained Linear Coding. Sensors.

[47] Ahn H., Jun I., Seo K.Y., Kim E.K., Kim T.-I. (2022). Artificial Intelligence for the Estimation of Visual Acuity Using Multi-Source Anterior Segment Optical Coherence Tomographic Images in Senile Cataract. Front. Med..

[48] Pattemore M., Gilabert R. (2023). Using eye-tracking to measure cognitive engagement with feedback in a digital literacy game. Lang. Learn. J..

[49] Chen H.-C., Tzeng S.-S., Hsiao Y.-C., Chen R.-F., Hung E.-C., Lee O.K. (2021). Smartphone-Based Artificial Intelligence–Assisted Prediction for Eyelid Measurements: Algorithm Development and Observational Validation Study. JMIR mHealth uHealth.

[50] Thomas T., Hoppe D., Rothkopf C.A. (2022). Measuring the cost function of saccadic decisions reveals stable individual gaze preferences. J. Vis..

[51] Kasinidou M. (2023). AI Literacy for All: A Participatory Approach. Proceedings of the 2023 Conference on Innovation and Technology in Computer Science Education V. 2.

[52] (2024). Neurons. *Predict Tech Paper*; Denmark.

[53] (2024). Neurons. *Predict Datasheet*.

[54] Awadh F.H.R., Zoubrinetzky R., Zaher A., Valdois S. (2022). Visual attention span as a predictor of reading fluency and reading comprehension in Arabic. Front. Psychol..

[55] Lusnig L., Hofmann M.J., Radach R. (2023). Mindful Text Comprehension: Meditation Training Improves Reading Comprehension of Meditation Novices. Mindfulness.

[56] Kobayashi J., Kawashima T. (2019). Paragraph-based Faded Text Facilitates Reading Comprehension. Proceedings of the 2019 CHI Conference on Human Factors in Computing Systems.

[57] Giles F. (2014). ‘The magazine that isn’t’: The future of features online. TEXT.

[58] Henderson C.M., Steinhoff L., Harmeling C.M., Palmatier R.W. (2021). Customer inertia marketing. J. Acad. Mark. Sci..

[59] Ju-Pak K.-H. (1999). Content dimensions of Web advertising: A cross-national comparison. Int. J. Advert..

[60] Zanker M., Rook L., Jannach D. (2019). Measuring the impact of online personalisation: Past, present and future. Int. J. Hum. Comput. Stud..

[61] Bernard M., Baker R., Chaparro B., Fernandez M. (2002). Paging VS. Scrolling: Examining Ways to Present Search Results. Proc. Hum. Factors Ergon. Soc. Annu. Meet..

[62] Garett R., Chiu J., Zhang L., Young S.D. (2016). A Literature Review: Website Design and User Engagement. Online J. Commun. Media Technol..

[63] Murali R., Conati C., Azevedo R. (2023). Predicting Co-occurring Emotions in MetaTutor when Combining Eye-Tracking and Interaction Data from Separate User Studies. Proceedings of the LAK23: 13th International Learning Analytics and Knowledge Conference.

[64] Douneva M., Jaron R., Thielsch M.T. (2016). Effects of Different Website Designs on First Impressions, Aesthetic Judgements and Memory Performance after Short Presentation. Interact. Comput..

[65] Hasan L. (2017). Evaluating the Usability of Educational Websites Based on Students’ Preferences of Design Characteristics. Int. Arab. J. E-Technol. (IAJeT).

[66] Pettersson J., Falkman P. (2023). Intended Human Arm Movement Direction Prediction using Eye Tracking. Int. J. Comput. Integr. Manuf..

[67] Lavdas A.A., Salingaros N.A., Sussman A. (2021). Visual Attention Software: A New Tool for Understanding the “Subliminal” Experience of the Built Environment. Appl. Sci..

[68] Gheorghe C.-M., Purcărea V.L., Gheorghe I.-R. (2023). Using eye-tracking technology in Neuromarketing. Rom. J. Ophthalmol..

[69] Rvacheva I.M. (2023). Eyetracking as a Modern Neuromarketing Technology. Ekon. I Upr. Probl. Resheniya.

[70] Zdarsky N., Treue S., Esghaei M. (2021). A Deep Learning-Based Approach to Video-Based Eye Tracking for Human Psychophysics. Front. Hum. Neurosci..

[71] Pettersson J., Falkman P. (2021). Human Movement Direction Prediction using Virtual Reality and Eye Tracking. Proceedings of the 2021 22nd IEEE International Conference on Industrial Technology (ICIT).

[72] Kondak A. (2023). AGH University of Krakow the application of eye tracking and artificial intelligence in contemporary marketing communication management. Sci. Pap. Silesian Univ. Technol. Organ. Manag. Ser..

[73] Morozkin P., Swynghedauw M., Trocan M. (2017). Neural Network Based Eye Tracking. Proceedings of the Computational Collective Intelligence: 9th International Conference, ICCCI 2017.

[74] Stein N., Bremer G., Lappe M. (2022). Eye Tracking-based LSTM for Locomotion Prediction in VR. Proceedings of the 2022 IEEE Conference on Virtual Reality and 3D User Interfaces (VR).

[75] Vazquez E.E. (2019). Effects of enduring involvement and perceived content vividness on digital engagement. J. Res. Interact. Mark..

[76] Sibarani B. (2019). Cognitive Engagement and Motoric Involvement in Learning: An Experiment on the Effect of Interaction Story Game on English Listening Comprehension in EFL Context. Engl. Linguist. Res..

[77] Yeung A.S. (1999). Cognitive Load and Learner Expertise: Split-Attention and Redundancy Effects in Reading Comprehension Tasks With Vocabulary Definitions. J. Exp. Educ..

[78] Alhamad K.A., Manches A., Mcgeown S. (2022). The Impact of Augmented Reality (AR) Books on the Reading Engagement and Comprehension of Child Readers. Edinb. Open Res..

[79] Al-Samarraie H., Sarsam S.M., Alzahrani A.I. (2023). Emotional intelligence and individuals’ viewing behaviour of human faces: A predictive approach. User Model. User-Adapt. Interact..

[80] Santiago-Reyes G., O’Connell T., Kanwisher N. (2022). Artificial neural networks predict human eye movement patterns as an emergent property of training for object classification. J. Vis..

[81] Buettner R. (2013). Cognitive Workload of Humans Using Artificial Intelligence Systems: Towards Objective Measurement Applying Eye-Tracking Technology. KI 2013: Advances in Artificial Intelligence: Proceedings of the 36th Annual German Conference on AI, Koblenz, Germany, 16–20 September 2013.

[82] Williams J. (2024). Consumer Behavior Analysis in the Age of Big Data for Effective Marketing Strategies. Int. J. Strat. Mark. Pract..

